# Data on biosynthesis of BPAF glucuronide, enzyme kinetics of BPAF glucuronidation, and molecular modeling

**DOI:** 10.1016/j.dib.2018.12.033

**Published:** 2018-12-17

**Authors:** Darja Gramec Skledar, Jurij Trontelj, Johanna Troberg, Tihomir Tomašič, Anamarija Zega, Moshe Finel, Lucija Peterlin Mašič

**Affiliations:** a Faculty of Pharmacy, Aškerčeva 7, 1000 Ljubljana, Slovenia; b Division of Pharmaceutical Chemistry, Faculty of Pharmacy, University of Helsinki, Helsinki, Finland

## Abstract

Bisphenol AF (BPAF) is in the body mainly metabolized to the corresponding bisphenol AF glucuronide (BPAF-G). While BPAF-G is not commercially available, enzyme-assisted synthesis of BPAF-G using the human recombinant enzyme UGT2A1, purification of BPAF-G by solid phase extraction and semi-preparative HPLC and chemical characterization of BPAF-G by NMR and LC-MS/MS were performed and are described here. Furthermore, BPAF glucuronidation kinetics with the UGT enzymes that showed the highest glucuronidation activity in previous studies (i.e hepatic UGTs 1A3, 2B7, and 2B17, intestinal UGT 1A10 and UGT 2A1 that is present in airways) was performed and data is presented. Hepatic enzymes exhibited high affinities toward BPAF, while extrahepatic UGTs 2A1 and 1A10 showed the high v_max_ values (3.3 and 3.0 nmol/min/mg, respectively). To understand molecular interactions of BPA, BPAF and BPAF-G with ligand biding sites of several nuclear receptors, molecular modeling was performed and data on the binding modes of BPAF, BPA, and BPAF-G in the ligand-binding sites of nuclear receptors are presented.

This article is related to “Endocrine activities and adipogenic effects of bisphenol AF and its main metabolite” (Skledar et al., 2019).

**Specifications table**

**Table:** 

Subject area	Biosynthesis of glucuronides, molecular modeling, glucuronidation kinetics
More specific subject area	Biosynthesis of BPAF-G, BPAF glucuronidation kinetics by UGTs 1A3, 2B7, 2B17, 1A10, and 2A1, molecular modeling of BPAF, BPA, and BPAF-G to nuclear receptors
Type of data	Table, graph, figure
How data was acquired	*In-vitro* enzyme-assisted synthesis of BPAF-G using human recombinant enzyme UGT2A1 as the enzyme source. HPLC-UV analyses was performed using an Agilent 1100 series HPLC system (Agilent Technologies, Palo Alto, CA, USA), Varian DirectDrive 800 MHz, Bruker AVANCE III 400 MHz spectrometer, LC-MS/MS, Chem3D 16.0 (Chem3D 16.0, ChemOffice Professional 16.0 Suite, CambridgeSoft, OpenEye Scientific Software, Inc., Santa Fe, NM, USA.
Data format	Raw and analyzed
Experimental factors	BPAF-glucuronide was synthesized using the UGT2A1 (0.5 mg protein/mL) as enzyme source and UDPGA (1 mM) as cofactor at 37 °C for 24 h.
	BPAF glucuronidation kinetics were examined for UGTs 1A3, 2B7, 2B17, 1A10, and 2A1. Experiments were performed at 8 BPAF concentrations, ranging from 1–50 µM. The incubation times and protein concentrations varied from 10 min to 30 min and from 0.01–0.05 mg protein/ml, respectively.
Experimental features	*In-vitro* enzyme-assisted synthesis of BPAF-G using human recombinant enzyme UGT2A1 as the enzyme source; enzyme kinetics of BPAF glucuronidation by UGTs 1A3, 2B7, 2B17, 1A10, and 2A1; molecular modeling of BPAF, BPA and BPAF-G to nuclear receptors.
Data source location	University of Ljubljana
Data accessibility	Data is available within this article
Related research article	Skledar DG, Carino A, Trontelj J, Troberg J, Distrutti E, Marchianò S, Tomašič T, Zega A, Finel M, Fiorucci S et al. Endocrine activities and adipogenic effects of bisphenol AF and its main metabolite. Chemosphere. 2019 215:870-880 [1]

**Value of the data**

• The data show the optimized *In-vitro* enzyme-assisted synthesis of BPAF-G using the human recombinant enzyme UGT2A1 as the enzyme source.
• The data of enzyme kinetics show very high affinities of hepatic UGTs 2B7 and 2B17.
• The data show binding modes of BPA, BPAF and BPAF-G in the ligand-binding sites of different nuclear receptors
• The data will be important for better understanding of differences in endocrine activities among the tested compounds that were observed in previous *in vitro* and *in vivo* studies.
• These data will be important for better understanding of biotransformation and elimination of BPAF.

## 1 Data

**Fig. 1:**
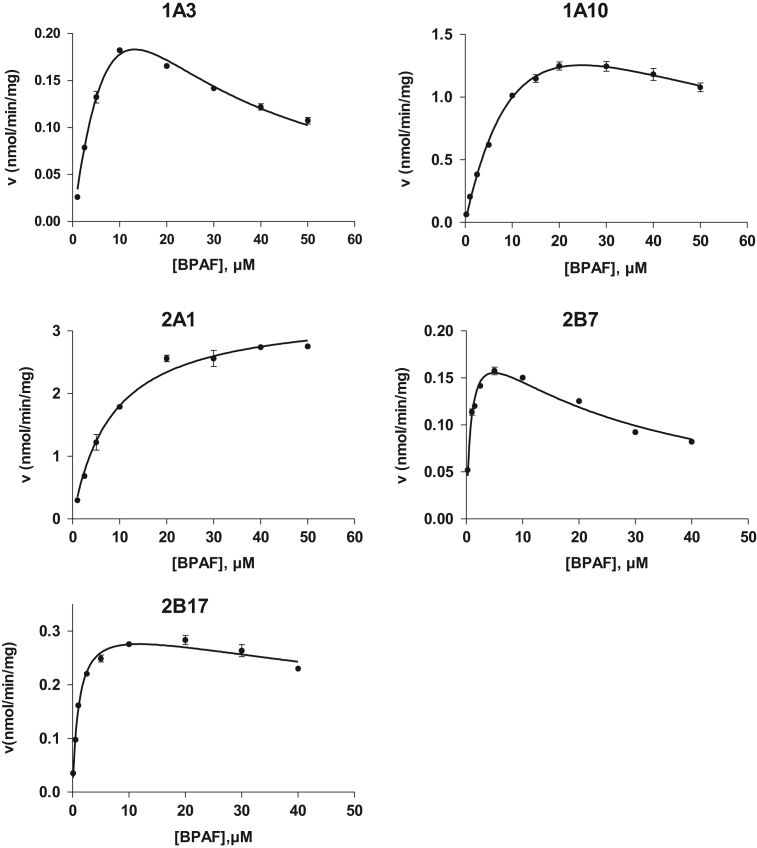
Enzyme kinetics of BPAF glucuronidation by UGTs 1A3, 1A10, 2A1, 2B7, and 2B17.

**Fig. 2:**
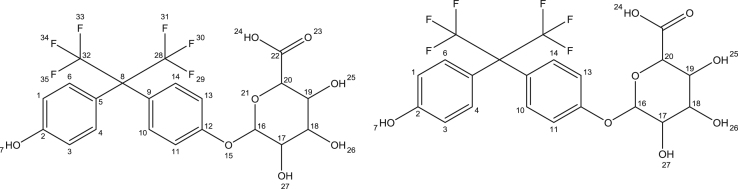

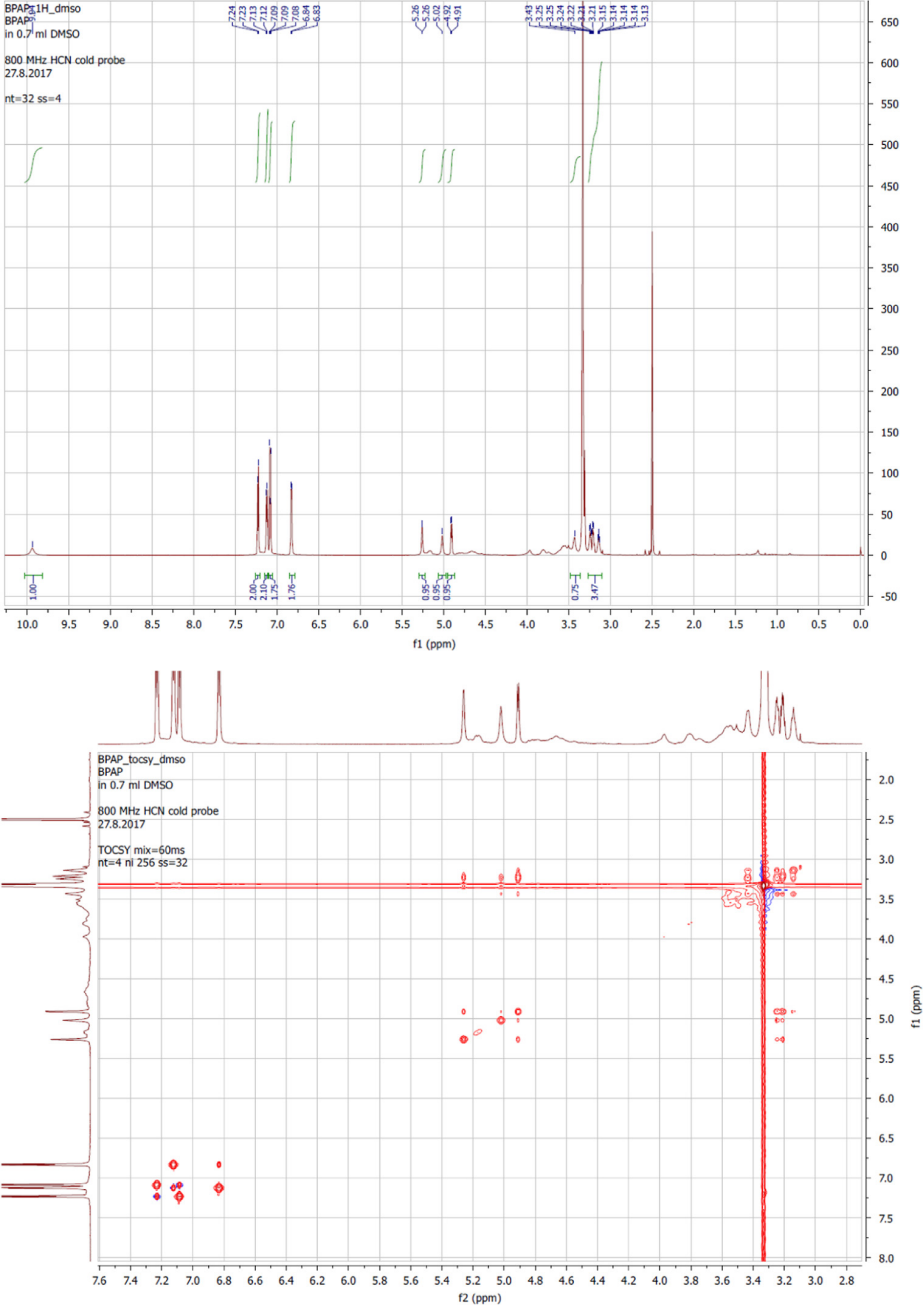
^1^H NMR and 2D TOCSY spectra were recorded at 25 °C on a Varian DirectDrive 800 MHz spectrometer equipped with a cryoprobe, in DMSO-*d*_*6*_ solution with TMS as an internal standard. 2D COSY spectra were recorded on a Bruker AVANCE III 400 MHz spectrometer in DMSO-*d*_*6*_solution, with TMS as internal standard. Data processing was performed with the MestReNova software. The ^1^H resonances were assigned based on characteristic chemical shifts and signal multiplicity of 1D proton spectra and ^1^H-^1^H correlations in 2D COSY and TOCSY. ^1^H NMR (800 MHz, DMSO-*d*_6_) δ 9.94 (s, 1H, H-7), 7.23 (d, *J* = 8.5 Hz, 2H, H-10,14), 7.12 (d, *J* = 8.4 Hz, 2H, H-4,6), 7.09 (d, *J* = 8.9 Hz, 2H, H-11,13), 6.83 (d, *J* = 8.5 Hz, 2H, H-1,3), 5.26 (d, *J* = 4.8 Hz, 1H, H-27), 5.02 (s, 1 H, H-26), 4.91 (d, *J* = 7.6 Hz, 1H, H-16), 3.47-3.40 (m, 1H, H-20), 3.28-3.18 (m, 2H, H-17,18), 3.17-3.12 (m, 1H, H-19).

**Fig. 3:**
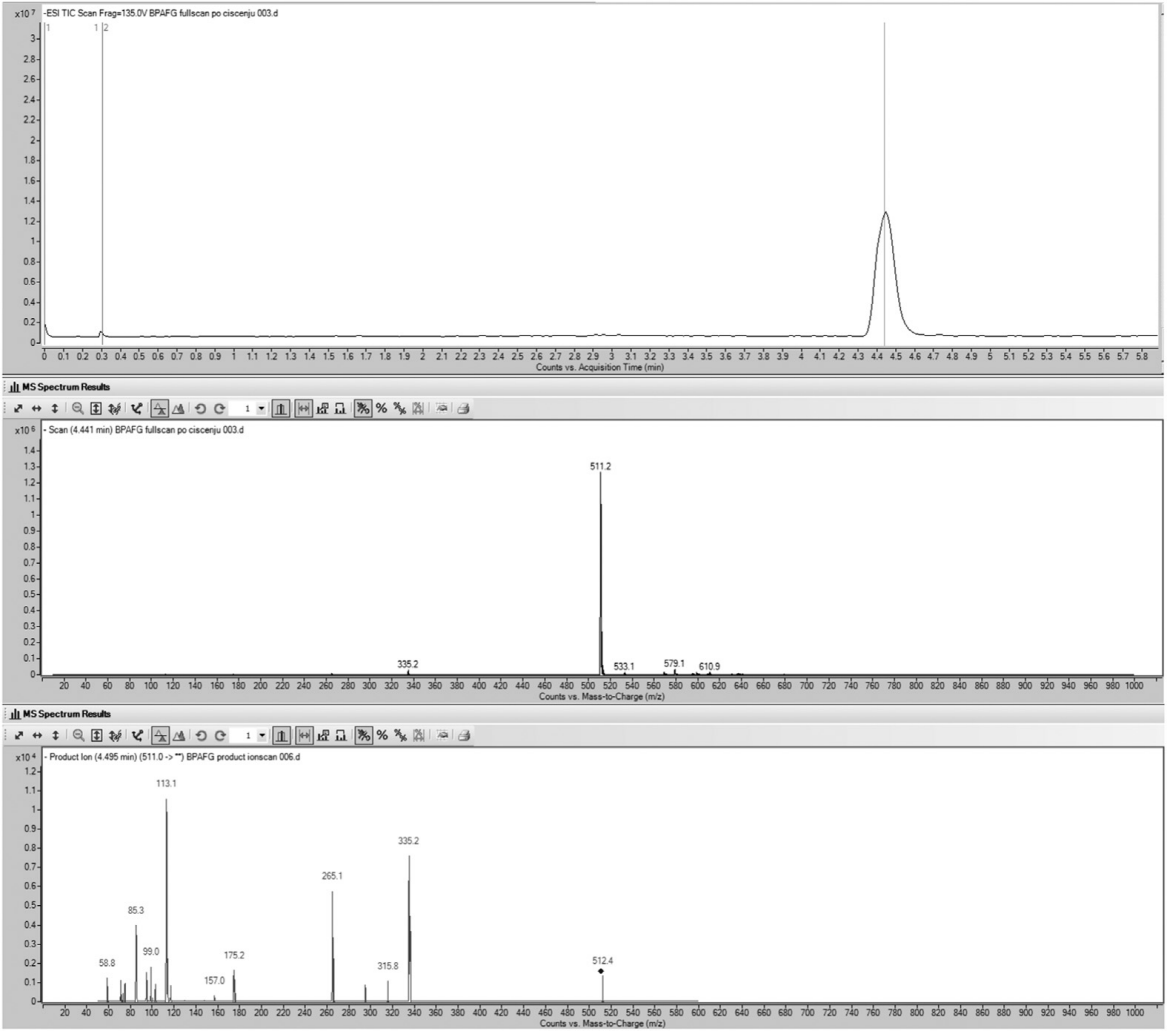
Chromatograms for LC-MS/MS characterization of BPAF-G. Top:Negative ion full-scan analysis of the purified pooled BPAF-G fraction and its corresponding MS spectrum (middle). Bottom: Product ion scan from precursor ion *m/z* 511 reveals all of the characteristic fragments of glucuronide (*m/z* 113, 175) and of BPAF (*m/z* 335, 265).

**Fig. 4:**
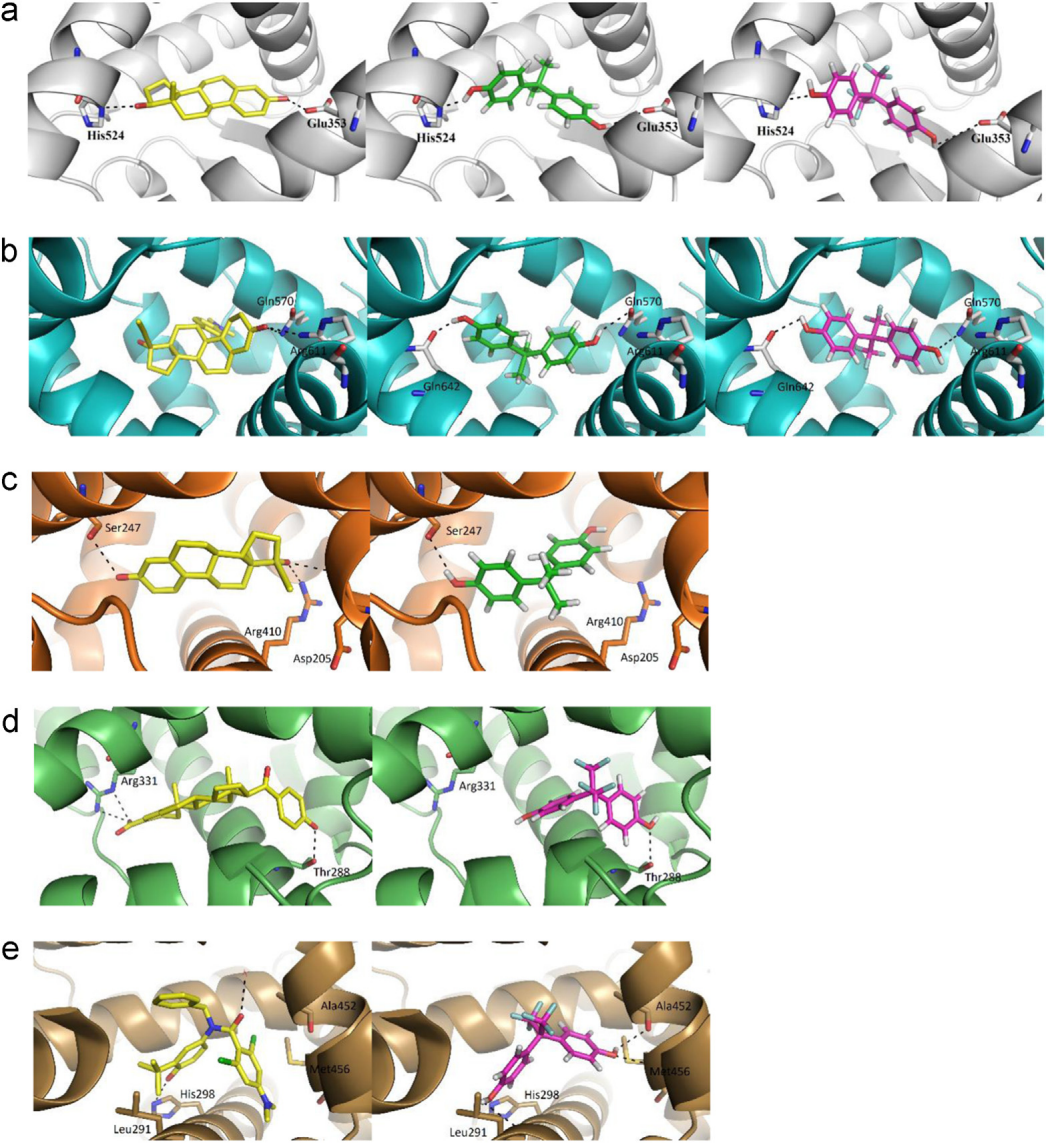
Docking binding mode of X-ray ligands (Table 2, yellow), BPA (green) and BPAF (magenta) in the ligandbinding sites of the estrogen receptor **(a)**(agonist-bound conformation; gray), glucocorticoid receptor **(b)**(antagonist-bound conformation; cyan), PXR **(c)**(agonist-bound conformation; orange), FXR **(d)**(agonist-bound conformation; green), and FXR **(e)**(antagonist-bound conformation; brown). The ligand is shown as a stick model, colored according to the atom types (blue, N; red, O; orange, S; green, Cl). For clarity, only the protein amino acids that form hydrogen bonds with the ligands are shown as sticks. Hydrogen bonds are indicated by black dotted lines.

The present data provide enzyme kinetics curves and obtained kinetics parameters for BPAF glucuronidation by UGTs 1A3, 1A10, 2A1, 2B7, and 2B17 (Fig. 1, Table 1). While BPAF-G is not commercially available, enzyme-assisted synthesis of BPAF-G using the human recombinant enzyme UGT2A1 was performed. Structural characterization of biosynthesized BPAF-G was performed by NMR and LC-MS/MS. Data presented 1H NMR and 2D TOCSY spectra of BPAF-G (Fig. 2) as well as representative LC-MS/MS chromatograms (Fig. 3). BPA, BPAF and BPAF-G showed agonistic and/or antagonistic activities toward some of the nuclear receptors [1]. The binding modes of the active bisphenols in the ligand-binding sites of these nuclear receptors as agonist-bound and antagonist-bound conformations at the molecular level were investigated using molecular docking and the data is presented here (Table 2, Fig. 4). For the androgen and thyroid receptors, and for PXR and PPARγ, the only crystal structures available were in complex with agonists, and therefore docking of the ligands to the potential antagonist-bound conformations of these receptors could not be performed.

**Table 1:** Kinetic parameters for BPAF glucuronidation by UGTs 1A3, 1A10, 2A1, 2B7, and 2B17.

**Enzyme**	**Model**	**V**_**max**_**(nmol/min/mg)**	**K**_**m**_**(µM)**	**r**^**2**^	**K**_**si**_
2A1	MM	3.3 ± 0.1	8.4 ± 0.8	0.98	
1A10	SI	3.0 ± 0.4	18 ± 3	0.99	35 ± 8
2B7	SI	0.21 ± 0.01	0.9 ± 0.1	0.96	27 ± 3
2B17	SI	0.33 ± 0.01	1.1 ± 0.1	0.98	123 ± 25
1A3	SI	0.79 ± 0.17	22 ± 6	0.98	8.0 ± 2.1

**Table 2:** Selected crystal structures of nuclear receptors used in molecular modeling studies.

**Receptor**	**Bound ligand**	**PDB code**	**Reference**
Estrogen	Estradiol^a^	1A52	[2]
	4-Hydroxytamoxifen^b^	2JF9	[3]
Androgen	Dihydrotestosterone a	3L3X	[4]
Thyroid	Triiodothyronine^a^	3GWS	[5]
Glucocorticoid	Cortisol^a^	4P6X	[6]
	Mifepristone^b^	3H52	[7]
PXR	17α-Ethinylestradiol^a^	4×1F	[8]
FXR	MFA-1^a^	3BEJ	[9]
	N-Benzyl-N-(3-(tert-butyl)-4-hydroxyphenyl)-2,6-dichloro-4-(dimethylamino) Benzamide^b^	4OIV	[10]
PPARγ	Tetrachlorobisphenol A^a^	3OSI	[11]

a agonist;

b antagonist

Bisphenol A and BPAF mimicked the binding mode of E2 in the ligand binding site of ER (Fig. 4A). The lipophilic central core of BPA and BPAF overlaps with the steroid core of the natural ligand, while the hydroxyl groups provide sites for additional hydrogen bond formation, which supports the observed agonistic activities of BPA and BPAF toward ER. The higher Chemgauss4 scoring function score for BPAF (-13.48) compared to BPA (-12.76) also corresponds to the observed differences in their *in-vitro* activities toward hERα, and is due to the more extensive hydrophobic interaction network of BPAF. As the agonist-bound ER is in a closed conformation, BPAF-G is too bulky to fit the binding site, which supports its lack of agonistic activity. In the antagonist-bound conformation of ER, the ligand binding site is larger, and there is enough space to accommodate all three of these bisphenols; however, they might not provide enough steric bulk, such as provided by tamoxifen, to prevent the conformational changes of ER that are necessary for its activation. In the case of the androgen, thyroid and glucocorticoid receptors, BPA and BPAF, but not BPAF-G, are predicted here to bind to the dihydrotestosterone-, T3-, and cortisol-binding sites, respectively. However, in comparison with the natural agonists, they appear not to offer enough steric and/or binding-interaction complementarity with the binding site to result in receptor activation. In contrast, BPA and BPAF show antagonistic activities against these three receptors. Among these, only the antagonist-bound conformation of the glucocorticoid receptor is available. Comparisons of mifepristone with BPA and BPAF in terms of their predicted binding modes (Fig. 4B) show that these bisphenols partially occupy the binding site to potentially prevent cortisol binding. However, BPA and BPAF do not form as many interactions (i.e., hydrogen bonds, hydrophobic interactions) as the potent antagonist mifepristone. The PXR (Fig. 4C), FXR (Fig. 4D, E) and PPARγ ligand-binding sites provide enough space for the binding of BPA, BPAF, and BPAF-G. As similar orientations and interactions were predicted for all three of these bisphenols, we cannot rationalise the observed differences in their activities based on these docking calculations.

## 2 Experimental design, materials and methods

### 2.1 Enzyme kinetics of BPAF glucuronidation by UGTs 1A3, 1A10, 2A1, 2B7, and 2B17

Glucuronidation activities of BPAF were tested in the recombinant human UGTs 1A3, 1A10, 2A1, 2B7, and 2B17. Selected experimental conditions are collected in Table 3. Briefly, the incubation mixtures contained phosphate buffer (50 mM, pH 7.4), MgCl_2_ (10 mM), 2% DMSO, BPAF at 8 different concentrations (1–50 µM) and UGT enzyme (Table 3) and were initiated by addition of UDPGA at the final concentration of 5 mM. Samples were incubated from 10–30 min at 37 °C and the reactions were terminated by the addition of 10 µL 70% perchloric acid and transferred to ice for 15 min. The tubes were then centrifuged at 16,000 *g* for 10 min and the supernatants were subjected to HPLC analysis. The HPLC analysis was performed as described previously [1].

**Table 3:** Experimental conditions for measuring BPAF glucuronidation kinetics.

**Enzyme**	**Substrate concentrations (µM)**	**Enzyme concentration (mg/mL)**	**Incubation times (min)**
UGT1A3	1, 2.5, 5, 10, 20, 30, 40, 50	0.01	20
UGT1A10	0.25, 1, 2.5, 5, 10, 15, 20, 30, 40, 50	0.01	10
UGT2A1	1, 2.5, 5, 10, 20, 30, 40, 50	0.01	10
UGT2B7	0.25, 1, 1.5, 2.5, 5, 10, 20, 30, 40	0.05	30
UGT2B17	0.1, 0.5, 1, 2.5, 5, 10, 20, 30, 40, 50	0.01	10

The data were analyzed using the GraphPad Prism 5.04 software for Windows (GraphPad Software Inc., San Diego, CA, USA), with the most appropriate kinetics model selected (i.e., Michaelis–Menten or substrate inhibition), as follows:

(1) $$\mathrm{Michaelis}-\mathrm{Menten:}\;v=\frac{V_{\max}\left[S\right]}{K_{m}\;+\;\left[S\right]}$$

(2) $$\mathrm{Substrate inhibition:}\;v=\frac{V_{\max}\left[S\right]}{K_{m}\;+\;\left[S\right](1+\frac{\left[S\right]}{K_{\mathrm{si}}})}$$

(*v*, velocity of the reaction; *v*_max_, maximal enzyme velocity; *S*, substrate concentration; *K*_m_, Michaelis–Menten constant; *K*_si_, constant of substrate inhibition).

### 2.2 Biosynthesis of bisphenol AF glucuronide

BPAF-glucuronide is not commercially available, and therefore we performed enzyme-assisted synthesis using the human recombinant enzyme UGT2A1 as the enzyme source. The incubation mixture had a final volume of 200 mL, and contained 50 mM phosphate buffer, pH 7.4, 10 mM MgCl_2_, UGT2A1 at a final concentration of 0.5 mg protein/mL, and the 0.1% bovine serum albumin (fatty acid free). BPAF was dissolved in DMSO and added in two equal aliquots (at the beginning, after 8 h) to a final concentration of 0.5 mM. The reaction mixture was preincubated for 5 min at 37 °C, and then the glucuronidation reaction was initiated by addition of UDPGA to a final concentration of 1 mM. The glucuronidation reaction was carried out for 24 h at 37 °C, and then terminated by addition of ice-cold 25% (v/v) acetonitrile, and transfer to −20 °C for 48 h. Following this, the mixture was centrifuged at 16,000×*g* for 10 min. The supernatant was collected, and subjected to solid phase extraction for cleaning and concentration of the samples. The aqueous phases of the incubation mixtures were loaded to the pre-conditioned (6 mL methanol, 6 mL water) cartridges. Each cartridge was then washed with 6 mL water and dried under vacuum. Elution was performed with 10 mL methanol. The methanol was evaporated under reduced pressure, and the solid residue was reconstituted in 20 mL 20% acetonitrile and subjected to semi-preparative HPLC. The glucuronide fractions were pooled, the acetonitrile was removed under reduced pressure, and the water was removed by lyophilisation. The resulting BPAF-G was characterized using both LC-MS/MS and ^1^H-nuclear magnetic resonance (NMR).

### 2.3 Molecular modeling of BPAF, BPA and BPAF-glucuronide, ligand and protein preparation, and ligand docking

Three-dimensional models of BPA, BPAF and BPAF-G were built usingChem3D 16.0 (Chem3D 16.0, ChemOffice Professional 16.0 Suite, CambridgeSoft). The geometries and charges of the molecules were optimized using the MMFF94 force-field, and partial atomic charges were assigned in Chem3D 16.0 [12]. The energy was minimized until the gradient value was smaller than 0.001 kcal / (mol×A°). The optimized structure was further refined with the GAMESS interface in Chem3D 16.0 (ChemOffice Professional 16.0 Suite, CambridgeSoft), using the semi-empirical PM3 method, the QA optimization algorithm, and GasteigerHückel charges for all of the atoms, for 100 steps. A library of conformers of BPA, BPAF and BPAF-G was generated using the OMEGA software (release 2.5.1.4, OpenEye Scientific Software, Inc., Santa Fe, NM, USA; www.eyesopen.com) using the default settings, which resulted in a maximum of 200 conformers per ligand [13].

For docking with the FRED software (Release 3.2.0.2, OpenEye Scientific Software, Inc., Santa Fe, NM, USA; www.eyesopen.com), the proteins (Table 2) were prepared using MAKE RECEPTOR (Release 3.2.0.2, OpenEye Scientific Software, Inc., Santa Fe, NM, USA; www.eyesopen.com) [14], [15], [16]. The grid box around the ligand bound in the crystal structure was generated automatically and was not adjusted, which resulted in different grid box sizes depending on the bound ligand. For ‘Cavity detection’, the slow and effective ‘Molecular’ method was used for the detection of binding sites. The inner and outer contours of the grid box were also calculated automatically using the ‘Balanced’ settings for the ‘Site shape potential’ calculation, which again resulted in different outer contour sizes that depended on the bound ligand. The inner contours were disabled and no constraints were used for the docking calculations.

Bisphenol A, BPAF and BPAF-G were docked using FRED (Release 3.2.0.2. OpenEye Scientific Software, Inc., Santa Fe, NM, USA; www.eyesopen.com) [14], [15], [16]. The docking resolution was set to high, with the other settings set as the defaults. Ten docking solutions were inspected visually, and the best-ranked FRED-calculated conformation was used for analysis and representation.

## References

[1] Skledar D.G., Carino A., Trontelj J., Troberg J., Distrutti E., Marchianò S., Tomašič T., Zega A., Finel M., Fiorucci S. (2019). Endocrine activities and adipogenic effects of bisphenol AF and its main metabolite. Chemosphere.

[2] Tanenbaum D.M., Wang Y., Williams S.P., Sigler P.B. (1998). Crystallographic comparison of the estrogen and progesterone receptor׳s ligand binding domains. Proc. Natl. Acad. Sci. USA.

[3] Heldring N., Pawson T., McDonnell D., Treuter E., Gustafsson J.A., Pike A.C. (2007). Structural insights into corepressor recognition by antagonist-bound estrogen receptors. J. Biol. Chem..

[4] Zhou X.E., Suino-Powell K., Ludidi P.L., McDonnell D.P., Xu H.E. (2010). Expression, purification and primary crystallographic study of human androgen receptor in complex with DNA and coactivator motifs. Protein Expr. Purif..

[5] Nascimento A.S., Dias S.M., Nunes F.M., Aparício R., Ambrosio A.L., Bleicher L., Figueira A.C., Santos M.A., de Oliveira Neto M., Fischer H. (2006). Structural rearrangements in the thyroid hormone receptor hinge domain and their putative role in the receptor function. J. Mol. Biol..

[6] He Y., Yi W., Suino-Powell K., Zhou X.E., Tolbert W.D., Tang X., Yang J., Yang H., Shi J., Hou L. (2014). Structures and mechanism for the design of highly potent glucocorticoids. Cell Res..

[7] Schoch G.A., D׳Arcy B., Stihle M., Burger D., Bär D., Benz J., Thoma R., Ruf A. (2010). Molecular switch in the glucocorticoid receptor: active and passive antagonist conformations. J. Mol. Biol..

[8] Delfosse V., Dendele B., Huet T., Grimaldi M., Boulahtouf A., Gerbal-Chaloin S., Beucher B., Roecklin D., Muller C., Rahmani R. (2015). Synergistic activation of human pregnane X receptor by binary cocktails of pharmaceutical and environmental compounds. Nat. Commun..

[9] Soisson S.M., Parthasarathy G., Adams A.D., Sahoo S., Sitlani A., Sparrow C., Cui J., Becker J.W. (2008). Identification of a potent synthetic FXR agonist with an unexpected mode of binding and activation. Proc. Natl. Acad. Sci. USA.

[10] Xu X., Liu P., Zhu Z.Y., Chen J., Fu H.A., Chen L.L., Hu L.H., Shen X. (2015). Structural basis for small molecule NDB (N-benzyl-N-(3-(tert-butyl)-4-hydroxyphenyl)-2,6-dichloro-4-(dimethylamino) Benzamide) as a selective antagonist of farnesoid X receptor α (FXRα) in stabilizing the homodimerization of the receptor. J. Biol. Chem..

[11] Riu A., Grimaldi M., le Maire A., Bey G., Phillips K., Boulahtouf A., Perdu E., Zalko D., Bourguet W., Balaguer P. (2011). Peroxisome proliferator-activated receptor γ is a target for halogenated analogs of bisphenol A. Environ. Health Perspect..

[12] Halgren T. (1996). Merck molecular force field .1. Basis, form, scope, parameterization, and performance of MMFF94. J. Comput. Chem..

[13] Hawkins P.C., Skillman A.G., Warren G.L., Ellingson B.A., Stahl M.T. (2010). Conformer generation with OMEGA: algorithm and validation using high quality structures from the Protein Databank and Cambridge Structural Database. J. Chem. Inform. Model.

[14] McGaughey G.B., Sheridan R.P., Bayly C.I., Culberson J.C., Kreatsoulas C., Lindsley S., Maiorov V., Truchon J.F., Cornell W.D. (2007). Comparison of topological, shape, and docking methods in virtual screening. J. Chem. Inform. Model.

[15] McGann M. (2011). FRED pose prediction and virtual screening accuracy. J. Chem. Inform. Model.

[16] McGann M.R., Almond H.R., Nicholls A., Grant J.A., Brown F.K. (2003). Gaussian docking functions. Biopolymers.

