# Supplementary material for: Data on biosynthesis of BPAF glucuronide, enzyme kinetics of BPAF glucuronidation, and molecular modeling
Source: Data Brief. 2018 Dec 17;22:977–86. doi: 10.1016/j.dib.2018.12.033 (PMC6356001; doi:10.1016/j.dib.2018.12.033)
Supplement: Supplementary file 1 — Supplementary material [file mmc1.doc]

Conflict of Interest and Authorship Conformation Form

Please check the following as appropriate:

- All authors have participated in (a) conception and design, or analysis and interpretation of the data; (b) drafting the article or revising it critically for important intellectual content; and (c) approval of the final version.
- This manuscript has not been submitted to, nor is under review at, another journal or other publishing venue.
- The authors have no affiliation with any organization with a direct or indirect financial interest in the subject matter discussed in the manuscript
- The following authors have affiliations with organizations with direct or indirect financial interest in the subject matter discussed in the manuscript:

Author’s name Affiliation

On behalf of all authors: corresponding author prof. dr. Lucija Peterlin Mašič
